# Serum levels of inflammatory mediators as prognostic biomarker in silica exposed workers

**DOI:** 10.1038/s41598-021-92587-0

**Published:** 2021-06-25

**Authors:** José Jesús Blanco-Pérez, Sara Blanco-Dorado, Javier Rodríguez-García, Mª Elena Gonzalez-Bello, Ángel Salgado-Barreira, Adriana Carolina Caldera-Díaz, Abel Pallarés-Sanmartín, Alberto Fernandez-Villar, Francisco Javier González-Barcala

**Affiliations:** 1 Department of Pneumonology, University Hospital Complex of Vigo, Pontevedra, Spain; 2 IRIDIS Group (Investigation in Rheumatology and Immuno-Mediated Diseases) Galicia Sur Health Research Institute (IIS Galicia Sur), Vigo, Spain; 3 Department of Pharmacy, University Hospital Complex of Santiago de Compostela, Santiago de Compostela, Spain; 4 Department of Clinical Analysis, University Hospital Complex of Santiago de Compostela (CHUS)-SERGAS, Santiago de Compostela, Spain; 5 Instituto de Investigación Sanitaria (IDIS), Santiago de Compostela, Spain; 6 Department of Pneumonology, University Hospital Complex of Vigo, Pontevedra, Spain; 7 Methodology and Statistics Unit, Galicia Sur Health Research Institute (IIS Galicia Sur), Vigo, Spain; 8 Department of Radiology, University Hospital Complex of Vigo, Pontevedra, Spain; 9 Department of Pneumonology, University Hospital Complex of Santiago de Compostela, Spanish Biomedical Research Networking Centre-CIBERES, Santiago de Compostela, Spain

**Keywords:** Health occupations, Medical research

## Abstract

Silicosis is a diffuse interstitial lung disease caused by sustained inhalation of silica and silicates. Several cytokines are activated by their inhalation and can mediate the process of pulmonary fibrosis. The identification of biomarkers could allow an early diagnosis before the development of radiological alterations and help monitor the evolution of patients. The objetive of this study was to determine the clinical significance of specific biomarkers, to estimate their association with the development, severity and/or progression of silicosis, and identify determinants of this evolution. We conducted a prospective observational study in patients attending the pulmonology clinic from 2009 to 2018. Serum levels of the following inflammatory mediators were assessed: interleukin-6 (IL-6), interleukin 2 receptor subunit alpha (IL2R) interleukin 1 beta (IL1B), interleukin-8 (IL-8), tumour necrosis factor-alpha (TNF-α), transforming growth factor-beta1 (TGF-β1), alpha-1 antitrypsin (AAT), C-reactive protein (CRP), lactate dehydrogenase (LDH) and ferritin in subjects exposed to silica, with and without silicosis. Association between those inflammatory mediators with lung function measurements and radiological severity of disease and their impact on prognosis were analysed. 337 exposed to silica (278 with silicosis) and 30 subjects in the control group were included. IL-8, α1AT, ferritin, CRP and LDH levels were higher in silicosis than in those exposed to silica without silicosis. IL-8, LDH and AAT levels were associated with progression of silicosis and IL-6, IL-8, LDH, AAT, ferritin, and CRP with vital status. The results of the ROC analysis indicated the potential of IL-8 as a biomarker in the presence of silicosis and for the prediction of mortality.

## Introduction

Silicosis is a chronic irreversible interstitial pulmonary disease caused by repeated inhalation of crystalline silica dust. Silicosis remains a problem in developing countries, due to lack of preventive measures, but it also remains a major occupational health concern in developed countries, having an important economic and social impact on workers and the healthcare system^1,2^. Silica exposure has been associated with diverse pathologies in addition to silicosis, such as the increased risk of tuberculosis and nontuberculous mycobacterial disease, chronic obstructive pulmonary disease, lung cancer, chronic kidney disease and systemic autoimmune rheumatic disease (SARD)^3,4^. A diagnosis of silicosis is based on the combination of a history of silica dust exposure and matching radiological findings, along with the exclusion of other entities, such as tuberculosis, fungal infections, sarcoidosis, idiopathic pulmonary fibrosis and other interstitial pulmonary diseases. Silicosis usually manifests itself in simple and complicated chronic forms. These forms generally appear after 10–15 years of exposure and symptoms range from asymptomatic simple chronic silicosis detected on radiological imaging to complicated silicosis, which frequently presents with dyspnoea and cough^5^.

Although the molecular mechanisms in the pathogenesis of silicosis are still not entirely known, the phagocytosis of crystalline silica causes an active inflammation of the phagosomes, followed by a phagosomic destabilisation that releases contents into the cytosol, with the corresponding activation of the resulting active enzyme complex (NALP3/inflammasome). Eventually, caspase-1 activates cytokines IL-1β and IL-18^4,6^, which engage fibroblasts to build up layers of collagen fibers and trigger the inflammatory cascade, including pro-inflammatory cytokines such as IL-6, IL-8, transforming growth factor (TGF-β), tumor necrosis factor (TNF–α) and others^7^. Currently, the diagnosis of silicosis includes detection of radiological abnormalities, which are late and demonstrate irreversible disease. Once the disease is contracted, silicosis can evolve to cause important morbidity and mortality, even after exposure is ceased. To date, different potential biomarkers have been suggested^8^ but the lack of definitive results precludes use in clinical practice.

The objective of our study was to determine the clinical significance of specific biomarkers, in order to estimate their effect on the development, severity and/or progression of silicosis.

## Methods

We conducted a prospective cohort study including subjects with history of at least 5 years of occupational silica dust exposure and with diagnosis of silicosis according to the guidelines of the Spanish Society of Pulmonology and Thoracic Surgery (SEPAR)^5^ between January 2009 to December 2018. Also, a control group consisting of hospital workers unexposed to silica, whose smoking history and age was similar to the subjects in the cohort, was included. Individuals presenting with acute disease in the previous three months, individuals diagnosed with sarcoidosis, and those whose samples could not be collected and those who refused to participate, were excluded.

The study was approved by the clinical research ethics committee of Galicia (Comité de Ética de Investigación de Galicia) and was carried out according to the principles of the declaration of Helsinki, as well as the current biomedical research legislation. Written informed consent from all participants was obtained prior to their inclusion in the study.

Demographic characteristics, body mass index (BMI), smoking history, degree of dyspnoea (MRC scale), co-morbidity measured by the Charlson Index^9^, and a ferritin blood test, CRP, LDH and AAT, were collected for all the subjects included in the study.

During the prospective follow-up, over at least 2 years clinical, functional and radiological assessments were gathered. The onset of the disease in healthy exposed patients and the disease progression in subjects previously diagnosed with silicosis were analysed using chest X-ray images and/or a computerized tomography (CT). Also, visits to the emergency department, hospital admissions due to respiratory causes and mortality were also assessed.

The chest X-ray and CT scan were interpreted by radiologists with extensive experience in silicosis. Small opacities were classified in four different categories: (0) absence of small opacities (1) scanty small opacities; (2) abundant small opacities without vascular blurring; (3) highly abundant small opacities with scarcely visible normal lung anatomical structures^10^. Large opacities were classified as follows: category A, one or more opacities greater than 10 mm and less than 1/4 of the CT right side area (quadrant) at the level of the carina; category B, one opacity greater than “A” but less than 1/2 of the CT right side area (two quadrants) at the level of the carina; category C, one opacity or the addition of all the opacities constituting more than half of the CT right side area at the level of the carina^11^. Any progression, regardless of the reached values of nodular profusion, was interpreted as radiological progression, and the passage from simple silicosis to complicated silicosis (A, B or C), from complicated silicosis A to B or C, or from B to C, was interpreted as progression with category change.

Spirometry and other tests measuring pulmonary function and diffusing capacity of the lungs (DLCO) were conducted following the *Spanish Society of Pulmonology and Thoracic Surgery* (SEPAR) and the *European Respiratory Society* (ERS) recommendations^12,13^. Forced vital capacity (FVC), forced expiratory volume in one second (FEV1), FEV1/FVC ratio, total lung capacity (TLC) and diffusing capacity for carbon monoxide (DLCO) were analysed. A FEV1/FCV ratio < 70 was considered obstructive airflow limitation, and restrictive airflow limitation if the TLC was < 80%^11^. A 6-min test was conducted according to the American Thoracic Society guidelines^14^.

For the biomarker measurement, plasma samples from the patients were separated from venous blood and stored frozen at − 80 °C until analysis. The following cytokines were determined: IL1-beta, IL2R, IL6, IL8, TNF-alpha and TGF-beta. The biomarkers quantitative determination was carried out as follows:

Measurement of TGF-ß. TGF-ß was determined through an ELISA (Enzyme-linked immunosorbent assay) distributed by R&D Systems Inc. (Minneapolis, USA). As a prior step to the analysis, samples were treated to transform TGF-ß in its immunoreactive form in the following way: (i) a 40 µl serum aliquot was mixed with 20 µl of a 1 N HCl solution, and the mixture was then stirred and incubated for 10 min at room temperature; (ii) acidified samples were neutralized through the addition of 20 µl of a 1,2 N NaOH/0,5 M HEPES previously prepared solution; (iii) finally, prior to analysis, samples were diluted 1:20 as per indicated procedure, following the instructions of the manufacturer.

Measurement of IL-6, IL-8, IL-1ß, IL-2R and TNF-α: two different analysers were used to measure these cytokines: INMULITE 1000 (IL2-R, IL-8 and TNF) and INMULITE 2000 (IL-6 and IL-2R) (Siemens Healthcare GmbH, Erlangen, Germany). Both analysers conduct a solid phase sequential enzyme immunometric assay by chemiluminescence. To measure the different samples, reagents provided by the manufacturer were used in both devices. Recommendations concerning calibration, adjustment and quality control were followed in order to obtain an optimal performance of the equipment.

The results of the qualitative variables were represented as frequency and percentage, while quantitative variables were represented as mean and standard deviation. Comparisons were made of patients’ baseline characteristics, pulmonary function tests and different biomarkers between controls of subjects exposed to silica and subjects with simple or complicated silicosis. The chi-squared test was used for the comparison of qualitative variables, and ANOVA testing for quantitative variables. When means are not equal Bonferroni post hoc tests have been carried out to determine which means presented statistically significant differences.

With the variables and biomarkers showing significant association with patient survival multivariate Cox regression analyses were conducted, and with progression, multivariate logistic regression analyses were used, in order to determine those variables showing an independent association with these two prognostic variables. ROC curves were used and the AUC of the biomarkers that showed greater association with progression and mortality was measured in order to estimate the predictive ability of biomarkers. Statistically significant differences were taken at the level of p < 0.05.

## Results

337 male subjects with an average age of 50,4 (± 11,2) years were included in the biomarker cohort. 59 were exposed to silica without silicosis (16%), 129 (35%) diagnosed with simple silicosis and 149 (40,6%) diagnosed with complicated silicosis. With regard to the work history, 274 participants (74,7%) worked in granite quarries, 40 (10,9%) were ornamental stone cutters, and 22 (6,5%) were engaged in other works, such as stone house building, coal mining, stone workers exposed to dust generated from the handling of quartz conglomerates, ceramic workers, aggregates and dental technicians. The average time of exposure to silica dust was 24,1 years, and the follow-up covered 6–7 years, during which 65 (19,3%) patients passed away.

Tables 1 and 2 show the most significant demographic, clinical, functional and prognostic characteristics of patients.

**Table 1:** General characteristics of the patients included in the study.

	All (n = 367)	Exposed (n = 59)	Simple silicosis (n = 129)	Complicated silicosis (n:149)	**p**
Age; average (SD)	50.4 (11.2)	46.2 (13.6)	49.7 (9.8)	52.6 (10.7)	0.001**
**Smoking**					
Active smoker	79 (23.5)	24 (40.7)	35 (27.3)	20 (13.4)	0.001**
Former smoker	157 (46.7)	18 (30.5)	56 (43.8)	83 (55.7)	
Non-smoker	100 (29.8)	17 (28.8)	37 (28.9)	46 (30.9)	
BMI; average (SD)	27.9 (4.0)	29.9 (4.9)	28.4 (3.6)	26.8 (3.7)	< 0.001**
COPD	67 (20.0)	2 (3.4)	18 (14.1)	47 (31.8)	< 0.001**
Asthma	31 (9.3)	8 (14.0)	16 (12.6)	7 (4.7)	0.032*
Charlson Index; average (SD)	0.98 (1.4)	0.74 (1.4)	0.86 (1.4)	1.18 (1.4)	0.062
Duration of exposure	24.1 (9.4)	21.2 (9.2)	25.5 (9.3)	23.9 (9.5)	0.031*
**Work**					
Quarry	274 (74.7%)	38 (64.4%)	105 (82.0%)	131 (87.9%)	< 0.001*
Construction	40 (10.9%)	18 (30.5%)	12 (9.4%)	10 (6.7%)	
Others	22 (6.5%)	3 (5.1%)	11 (8.6%)	8 (5.4%)	
PH	16 (4.8%)	0	1 (0.8%)	15 (10.1%)	< 0.001**
SARD	40 (11.9%)	6 (10.2%)	17 (13.2%)	17 (11.4%)	0.817
Tuberculosis	54 (16.3%)	1 (1.8%)	21 (16.7%)	32 (21.5%)	0.003*
Death	65 (19.3%)	4 (6.8%)	13 (10.2%)	48 (32.2%)	< 0.001**

*BMI* body mass index, *PH* pulmonary hypertension, *SARD* systemic autoimmune rheumatic disese, *COPD* chronic obstructive pulmonary disease.

Statistical significance was determined using χ2 test and ANOVA test for qualitative and continuous variables, respectively.

*p < 0.05; **p < 0.001.

**Table 2:** Baseline clinical and lung function characteristics, change of lung function, and progression in patients.

	Expose (n = 59)	Simple silicosis (n = 129)	Complicated silicosis (n = 149)	p
Initial dyspnea MRC ≥ 2; n (%)	16 (27.1%)	78 (60.5%)	118 (79.2%)	< 0.001
Initial 6-min test (meters); average (SD)	445.0 (73.8)	415.0 (100.9)	390.0 (84.9)	0.021
Initial FVC/FEV1 ratio; average (SD)	79.4 (8.3)	76.9 (9.2)	67.8 (13.1)	< 0.001
Initial TLC (expected%); average (SD)	90.6 (12.3)	86.5 (13.3)	77.1 (15.5)	< 0.001
Initial DLCO (expected%); average (SD)	88.5 (19.9)	85.5 (16.4)	70.5 (19.6)	< 0.001
FVC/FEV < 70 in follow-up; n (%)	8 (14.3%)	22 (17.3%)	73 (49.0%)	< 0.001
FVC > 10% decrease in follow-up; n (%)	8 (24.2%)	17 (15.2%)	40 (30.3%)	0.021
DLCO decrease in follow-up; n (%)	2 (9.1%)	19 (20.0%)	43 (36.8%)	0.003
Progression; n (%)	0 (0.0%)	39 (30.2%)	94 (63.1%)	< 0.001
Death; n (%)	4 (6.8%)	13 (10.2%)	48 (32.2%)	< 0.001

Statistical significance was determined using χ2 test and ANOVA test for qualitative and continuous variables, respectively.

*p < 0.05; **p < 0.001.

Progression (Table 2) was observed in 115 individuals during the follow-up based on X-ray criteria (0 exposed, 39 simple silicosis and 89 complicated silicosis), 123 individuals based on CT criteria (0 exposed, 39 simple silicosis and 84 complicated silicosis) and 52 individuals based on category change. Progression based on at least one of the different criteria occurred in 133 patients, 39,5% of cases (0 exposed, 39 simple silicosis and 94 complicated silicosis).

In addition to the serum samples of the 337 patients exposed to silica, 30 unexposed subjects from the control group were included in the analysis, with an average age of 52,4 (7,7).

Table 3 shows the average concentration of the selected biomarkers for the four groups: IL1B, IL2R, IL-6, IL-8, TNF-α, TGF-β1, LDH, α1AT, ferritin and CRP. As illustrated in the abovementioned table, patients exposed to silica and diagnosed with silicosis showed higher levels of IL2R, IL-6 and IL-8 than those corresponding to healthy controls. Likewise, patients diagnosed with silicosis showed higher levels of IL-8, AAT, CRP and LDH than exposed patients, and patients diagnosed with complicated silicosis showed higher levels of IL2R, IL-6 IL-8, AAT, ferritin, CRP and LDH than those diagnosed with simple silicosis. In the post hoc analysis of the concentrations of biomarkers in the 3 groups (exposed, simple silicosis and complicated silicosis) only IL-8 showed differences in the 3 groups and was included in the multivariate model. LDH, alpha 1- antitrypsin, ferritin and CRP showed significant differences between complicated silicosis and exposed and simple silicosis but not between exposed and simple silicosis, and IL-6 showed differences only between complicated and simple silicosis.

**Table 3:** Serum levels of the different biomarkers in control groups and silica-exposed patients. with and without silicosis.

	All	Healthy controls (n = 30)	Exposed (n = 59)	Simple silicosis (n = 129)	Complicated silicosis (n = 149)	p
IL1B	6.72 (12.2)	4.54 (1.4)	8.5 (25.2)	6.8 (9.0)	6.4 (7.0)	0.509
IL2R	428.9 (245.7)	315.9 (104.9)	424.9 (224.0)	424.3 (221.7)	457.2 (285.7)	0.038*
IL-6	7.8 (8.7)	3.2 (1.6)	7.2 (10.9)	6.8 (4.9)	9.9 (10.5)	< 0.001**
IL-8	34.5 (37.2)	14.3 (12.0)	18.4 (13.1)	32.5 (42.8)	46.6 (37.5)	< 0.001**
TNF-α	7.5 (17.1)	8.74 (9.4)	9.6 (26.9)	7.1 (15.8)	6.8 (14.2)	0.705
TGF-β1	20.5 (7.5)	24.11 (7.1)	20.3 (7.9)	20.5 (6.8)	19.8 (7.9)	0.041*
AAT	142.6 (36.2)	–	128.7 (36.6)	138.7 (32.6)	151.4 (37.0)	< 0.001**
LDH	378.5 (99.4)	–	342.9 (92.9)	358.2 (86.7)	410.3 (103.1)	< 0.001**
Ferritin	504.6 (491.5)	–	303.5 (236.8)	444.8 (303.7)	619.6 (633.0)	0.001**
CRP	10.5 (17.8)	–	6.87 (16.2)	7.5 (10.5)	14.5 (22.1)	0.001**

*IL1β* Interleukin 1 beta, *IL2R* interleukin 2 receptor subunit alpha, *IL-6* Interleukin 6, *IL-8* interleukin 8, *TNF-α* Tumour necrosis factor alpha, *TGF-β1* Transforming growth factor-beta 1, *AAT* alpha-1 antitrypsin, *LDH* lactate dehydrogenase, *CRP* C-reactive protein.

Statistical significance was determined using χ2 test and ANOVA test for qualitative and continuous variables; respectively.

*p < 0.05; **p < 0.001.

Regarding the differences in biomarkers according to the category of complicated silicosis, the levels of IL-8, LDH, alpha-1 antitrypsin (AAT) and ferritin were higher in the upper category within complicated silicosis (Table 4). As can be seen in Table 5, the levels of IL-8, LDH and AAT were associated with progression and those of IL-6, IL-8, LDH, AAT, ferritin and CRP with death.

**Table 4:** Serum levels of biomarkers in the group of complicated silicosis A, B and C.

	Complicated Silicosis A (n = 86)	Complicated silicosis B (n = 42)	Complicated silicosis C (n = 21)	p
IL1B	7.2 (8.8)	5.1 (2.3)	6.2 (3.3)	0.280
IL2R	491.8 (315.4)	406.4 (231.2)	417.5 (243.7)	0.225
IL-6	9.1 (9.7)	11.4 (13.7)	10.0 (5.0)	0.511
IL-8	35.6 (20.4)	50.2 (41.6)	84.4 (55.2)	< 0.001**
TNF-α	8.3 (18.4)	4.7 (2.7)	4.6 (2.2)	0.312
TGF-β1	19.3 (7.6)	20.1 (9.1)	21.3 (6.6)	0.557
LDH	385.9 (90.9)	413.0 (88.1)	503.6 (126.0)	< 0.001**
AAT	142.5 (38.2)	159.7 (32.4)	170.4 (30.6)	0.002*
Ferritin	554.2 (557.2)	490.7 (322.7)	1155.4 (1056.6)	0.001*
CRP	13.9 (24.4)	13.2 (16.9)	19.1 (21.2)	0.573

*IL1β* Interleukin 1 beta, *IL2R* interleukin 2 receptor subunit alpha, *IL-6* Interleukin 6, *IL-8* interleukin 8, *TNF-α* Tumour necrosis factor alpha, *TGF-β1* Transforming growth factor-beta 1, *AAT* alpha-1 antitrypsin, *LDH* lactate dehydrogenase, *CRP* C-reactive protein.

Statistical significance was determined using χ2 test and ANOVA test for qualitative and continuous variables, respectively.

*p < 0.05; **p < 0.001.

**Table 5:** Association between serum levels of biomarkers, progression and mortality.

	Progression		p	Death		p
	Yes	No		Yes	No	
IL-1	6.1 (4.3)	7.4 (16.0)	0.372	6.2 (4.4)	7.1 (14.0)	0.608
IL-2R	428.4 (258.5)	445.9 (248.4)	0.534	501.2 (312.4)	424.5 (234.1)	0.067
IL-6	8.7 (9.6)	7.9 (8.6)	0.429	11.1 (8.7)	7.5 (8.9)	0.004*
IL-8	44.7 (38.9)	30.7 (36.5)	0.001*	58.2 (50.4)	30.1 (29.1)	0.001*
TNF-α	7.9 (21.2)	7.0 (14.9)	0.669	7.6 (21.l)	7.3 (16.8)	0.896
TGF-β	20.5 (7.7)	19.9 (7.3)	0.493	19.7(7.2)	20.2 (7.5)	0.650
LDH	400.6 (107.1)	364.3 (91.5)	0.001*	445.5 (124.7)	363.4 (84.7)	< 0.001**
Alpha1AT	148.9 (35.6)	138.5 (36.1)	0.011*	173.2 (38.1)	135.7 (31.5)	< 0.001**
Ferritin	549.3 (519.3)	471.4 (469.0)	0.217	871.4 (866.4)	432.2 (335.4)	0.003*
CRP	12.1 (16.7)	9.5 (18.4)	0.202	20.9 (23.0)	8.0 (15.3)	< 0.001**

*IL1β* Interleukin 1 beta, *IL2R* interleukin 2 receptor subunit alpha, *IL-6* Interleukin 6, *IL-8* interleukin 8, *TNF-α* Tumour necrosis factor alpha, *TGF-β1* Transforming growth factor-beta 1, *AAT* alpha-1 antitrypsin, *LDH* lactate dehydrogenase, *CRP* C-reactive protein.

Statistical significance was determined using χ2 test and T Student test for qualitative and continuous variables, respectively.

*p < 0.05; **p < 0.001.

A multivariate Cox regression analysis was conducted taking into account the significant resulting variables from the univariate analysis. Variables associated with survival were age (HR = 1.03 [95% CI 1.00–1.05]), IL-8 (HR = 1.01 [95% CI 1.004–1.016]), LDH (HR = 1.003 [95% CI 1.001–1.003]), PCR (HR = 1.015 [95% CI 1.003–1.028]), Charlson Index > 3 (HR = 4.16 [95% CI 1.90–9.12]), MRC (HR = 2.00 [95% CI 1.05–3.80]) and progression (HR = 1.91 [95% CI 1.09–3.34]).

A multivariate logistic regression analysis was conducted to analyse the relationship between the different biomarkers and progression, no variables showed significance.

**Figure 1:**
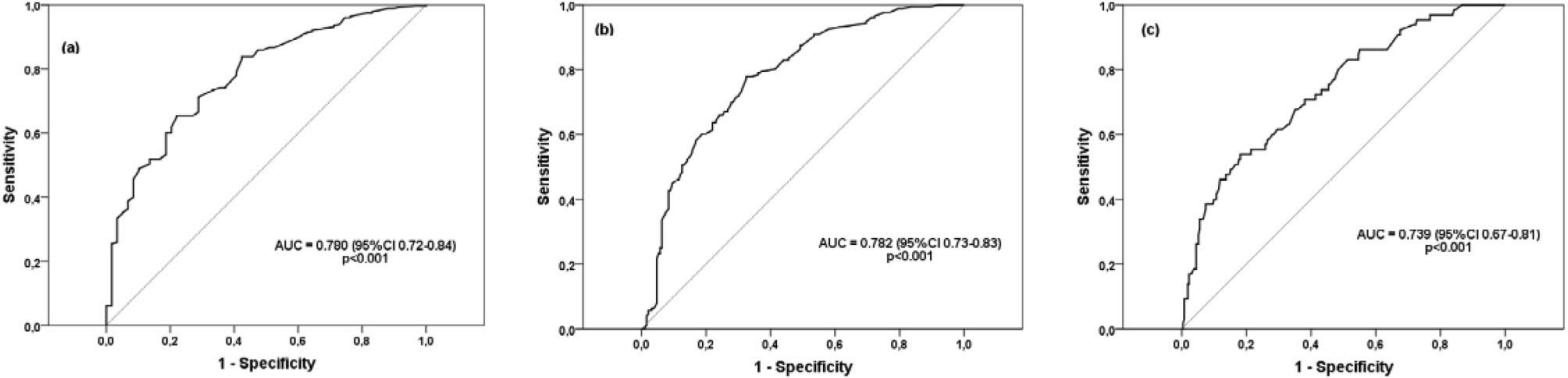
(**a**) ROC curve to show IL8 capacity to discriminate silicosis diagnosis from silica exposure. (**b**) ROC curve to show IL-8 capacity to discriminate complicated silicosis from the rest of silica exposed subjects. (**c**) ROC curve to show IL-8 capacity to discriminate mortality.

A ROC curve analysis was conducted, assessing the values of IL-8 to identify silicosis and predict mortality. Figure 1 show ROC curves that estimate the predictive capacity of the biomarker in silicosis identification. The AUC value for silicosis diagnosis was 0,780 (IC95% 0,717–0,842), and 0,782 (IC95%: 0,735–0,829) for the identification of complicated silicosis. The cut-off value of serum IL-8 for the final silicosis diagnosis was 17,2 pg/ml with sensitivity of 79%, specificity of 59%, positive predictive value of 90% and negative predictive value of 38%.

The ROC curve AUC to discriminate mortality with serum IL-8 was 0,739 (IC95%: 0,67–0,81). 20,2 pg/ml of serum IL-8 was determined as the optimal cut-off point, with a resulting sensitivity of 86%, specificity of 45%, positive predictive value of 27% and negative predictive value of 93%.

## Discussion and conclusions

This study is the first to analyse the clinical utility of a variety of serum biomarkers in such a wide cohort of silica dust exposed subjects. An overview of the main findings of this study shows that: (1) levels of IL-8, α1AT, ferritin, CRP and LDH were higher in patients diagnosed with silicosis than in those exposed to silica dust; (2) levels of IL2R, IL-6 IL-8, AAT, ferritin, CRP and LDH were higher in complicated silicosis than in simple silicosis; (3) levels of IL-8, LDH, AAT and ferritin rise as the disease severity increases (they are higher in patients diagnosed with silicosis than in those exposed to silica and in healthy controls, and they allow differentiation of patients with complicated silicosis from those with simple silicosis); (4) IL-8, LDH and AAT were associated with progression and IL-6, IL-8, LDH, α1AT, ferritin and CRP were associated with mortality; (5) independent association between survival and age, IL-8, dyspnoea, Charlson Index, LDH, PCR and progression; and (6) the results of diagnostic performance encourage the potential use of IL-8 as a biomarker in silicosis.

Previous research has described increased levels of inflammatory serum markers in silica exposed subjects^8,15^. It has been observed that the presence of different polymorphisms, alongside with TNF, IL-1RA and TGF-β1^16–18^ confer a higher risk of disease, and that levels of TNF-α show an increase before the development of a clinically recognised silicosis^19^ and that are associated with disease severity. Likewise, diverse studies associate TGF-β1 levels with disease progression and show that IL-2R can be a good indicator of silicosis-associated immunological alteration. The role of IL-8 is less clear; it seems to be unrelated to the risk of silicosis development, but related to progressive massive fibrosis (PMF)^20^.

Meanwhile, IL-6 is a multifunctional cytokine recognized as the main mediator in acute phase response with anti-inflammatory effects, exerting control on IL-1 and TNF-α production. Combined with TGF- β, IL-6 suppresses Treg generation and induces Th17 differentiation (a cytokine with pathogenic effect in different autoimmune diseases)^21^. Our study, similarly, to Braz et al.’s study^22^, shows higher IL-6 serum levels in patients with or without silicosis than in unexposed healthy subjects.

IL-1β is one of the mediators implied in the pathogenesis of silicosis, playing a crucial role in early inflammatory response^23^. In Leon et al.’s study^24^, which included 57 patients with silicosis and 18 controls, IL-1β was significantly higher in the complicated silicosis group than in healthy patients, but not so in relation to the simple silicosis group. Our study shows no differences between groups in IL-1β levels.

Views are divided concerning the behaviour of serum TNF, whose role is inducing influx of inflammatory cells, promoting secretion of other cytokines and potentiating the proliferation of fibroblasts and collagen deposits. Jiang et al.’s study^19^ shows that TNF-α plasma levels were significantly higher in 30 patients with silicosis than in 30 controls, and exposed workers without silicosis (28) presented higher levels than unexposed healthy subjects but lower than patients with silicosis. Thus, TNF-α seemed a strong candidate for an early biomarker. However, our series showed no differences in TNF-α levels between groups, regardless of silica exposure; and Barrett et al. appreciated diminution of TNF-a mRNA levels in studies in mice with cristobalite-induced silicosis^25^. Tomaz Braz et al.’s clinical work shows that while tumour necrosis factor receptor (TNFR1 y TNFR2) levels were associated with exposure and severity, this was not the case with TNF^26^, something the authors justified by the noting the instability of this cytokine, with a short life span that can vary throughout the day. Baird et al. reported the case of a patient who developed an accelerated silicosis with an anti-TNF-α treatment^27^. This case challenges the theory suggesting that increased TNF-α secretion acts as a possible mediator of disease progression and the fibrotic process in silicosis, and suggests more complex immunological interactions that are likely in the pathogenesis of fibrotic pulmonary diseases.

TGF-β has been accorded a key role in fibrosis, corroborated by its presence in histological sections of silicotic nodules**.** Lee et al.’s work shows higher TGF-β1 average levels in subjects with progressive silicosis than in those with no signs of progression and it was suggested that it could be considered as biomarker of progression^28^. Our study found no differences in TGF-β levels, just like León et al.’s research.

This can be explained because the fibrotic activity of TGF-β may depend on its concentration, with a stimulating effect at low concentrations of fibroblasts and an inhibitory effect at higher concentrations^29^. In higher concentrations, TGF-β decreases transcriptions and expression of platelet-derive growth factor (PDGF) receptor alpha subunits. Our study would confirm that TGF in low concentrations has stimulating effects on fibroblasts and an inhibitory effect in high concentrations. In fact, active TGF-β serum concentration is severely depressed in advanced atherosclerosis^30^. These differences could also be related to discrepancies between sample preparation methods, which may affect protein TGF-β1 levels detected in plasma.

Our study confirms of Lee et al.^20^ who noted the level of IL-8, a neutrophil chemotactic factor, was associated with the diagnosis of silicosis, the severity of silicosis and in our case, vital status too. Our results are in line with observations of increased levels of sIL-2R in patients with silicosis^31,32^. We observed that sIL-2R levels are decreased in healthy subjects compared to those diagnosed with silicosis, although there were no variations within the different categories of complicated silicosis. sIL-2R behaves as a good indicator for immunological alteration in silicosis^31^; even without clinical manifestations of any alteration in autoimmunity, sIL-2R correlates with ANA, anti-Scl-70 and anti-CENP-B titers, a statistically non-significant association in our series.

Unusually, a series of non-specific biomarkers such as CRP, AAT and LDH were assessed in our study on silicosis; we have not found any prior study in which ferritin has been analysed. However, several studies^33,34^ show elevated CRP levels, a non-specific biomarker, in patients exposed to silica and increasing according to silicosis severity, as was the case in our study. Our series shows elevated levels of alpha-1-AT, in a similar fashion to Montes II et al.^35^. The rise in plasma LDH concentration can be also detected in a variety of other processes, such as systemic infections or inflammations, muscle injuries, hemolysis, thromboembolism or malignancies. This rise has previously been assessed in silicosis in two prior studies, conducted in agate and denim sandblasting workers respectively. Both linked LDH plasma levels with silica exposure and severity^36,37^.

The presence of elevated serum ferritin is common in general medical practice in relation to alcohol consumption, metabolic syndrome, obesity, diabetes, hepatic disease, malignancy, infection and inflammation, behaving as an acute phase reactant in the latter; yet, serum ferritin had not been assessed in silicosis so far.

Our study has several limitations. While being a prospective study, no predefined time intervals were established for the follow-up and the realization of radiological or lung function studies. Therefore, the assessment of progression between tests could have been by differences in time interval. Other limitation is the lack of work place dust measurement, exposure is evaluated based on the exposure time, which is a relatively valid indicator, although less precise than the analysis of the individual exposure dose as cumulative exposure; besides, the necessity to standardise and optimise measurement methods must be taken into account^38^.

Another factor to be considered with regard to applicability of our results is the fact that our data come from a particular geographic area, and could relate to specific occupational exposures, work materials and climatic environments, limiting the external validity of our results elsewhere.

In conclusion, our results, based on a large number of samples, suggest that IL-8 has reasonable potential as a biomarker in the presence of silicosis and for the prediction of mortality.

Further research is needed on mediators of inflammation and fibrosis after silica exposure, including diverse cytokines which could allow us to differentiate silica dust inhalation from fibrotic disease progression and which could distinguish between the different categories of silicosis; nowadays, some of these cytokines have potential therapeutic application.
